# Identification of a novel set of genes reflecting different in vivo invasive patterns of human GBM cells

**DOI:** 10.1186/1471-2407-12-358

**Published:** 2012-08-17

**Authors:** Massimiliano Monticone, Antonio Daga, Simona Candiani, Francesco Romeo, Valentina Mirisola, Silvia Viaggi, Ilaria Melloni, Simona Pedemonte, Gianluigi Zona, Walter Giaretti, Ulrich Pfeffer

**Affiliations:** 1 IRCCS Azienda Ospedaliera Universitaria San Martino - IST - Istituto Nazionale per la Ricerca sul Cancro, Largo R. Benzi, 10, 16132 Genoa, Italy; 2 Dipartimento di Scienze della Terra, dell’Ambiente e della Vita (DISTAV), Università di Genova, Genoa, Italy; 3 TibMolbiol, Largo R. Benzi, 10, 16132, Genoa, Italy

## Abstract

**Background:**

Most patients affected by Glioblastoma multiforme (GBM, grade IV glioma) experience a recurrence of the disease because of the spreading of tumor cells beyond surgical boundaries. Unveiling mechanisms causing this process is a logic goal to impair the killing capacity of GBM cells by molecular targeting.

We noticed that our long-term GBM cultures, established from different patients, may display two categories/types of growth behavior in an orthotopic xenograft model: expansion of the tumor mass and formation of tumor branches/nodules (nodular like, NL-type) or highly diffuse single tumor cell infiltration (HD-type).

**Methods:**

We determined by DNA microarrays the gene expression profiles of three NL-type and three HD-type long-term GBM cultures. Subsequently, individual genes with different expression levels between the two groups were identified using Significance Analysis of Microarrays (SAM). Real time RT-PCR, immunofluorescence and immunoblot analyses, were performed for a selected subgroup of regulated gene products to confirm the results obtained by the expression analysis.

**Results:**

Here, we report the identification of a set of 34 differentially expressed genes in the two types of GBM cultures. Twenty-three of these genes encode for proteins localized to the plasma membrane and 9 of these for proteins are involved in the process of cell adhesion.

**Conclusions:**

This study suggests the participation in the diffuse infiltrative/invasive process of GBM cells within the CNS of a novel set of genes coding for membrane-associated proteins, which should be thus susceptible to an inhibition strategy by specific targeting.

Massimiliano Monticone and Antonio Daga contributed equally to this work

## Background

The Glioblastoma multiforme (GBM, stage IV Glioma) arise from neuroglial cells or their progenitors and represents the most aggressive brain tumor, with 15 months median survival after diagnosis, causing 4% of all cancer-related death despite recent improvement of diagnostic and treatment procedures. Surgery represents the standard treatment procedure. However, the vast majority of the patients affected by GBM experience a recurrence of the disease because of the spreading of cells beyond the limits of the resection [1]. The identification of the affected region of the central nervous system (CNS) to be resected is a major challenge. Neither advanced imaging techniques nor histological examination warrant against leaving some tumor cells in adjacent normal-looking brain tissue. Histologically normal brain tissue acquired at a distance greater than 4 cm from the GBM/Oligodendroglioma tumor was shown to give rise to tumor colonies in soft agar culture [2]. Therefore, the ability of GBM cells to invade the host tissue is one of the biological features of this disease that eventually has the most detrimental impact on the life expectancy of the patient [1]. In addition, these cells are difficult to eradicate since they invade areas of the CNS with an intact blood–brain barrier. As a consequence, the targeting of the GBM invasion process is a major topic of interest [3-5].

In the past years, some reports have focused on the inverse relationship between growth/apoptosis sensitivity and migration of glioma cells [6] and production by glioma cells of factors able to enhance invasion in an autocrine fashion [5]. Other research groups have shown the ability of the microenvironment to influence migration properties via cell-extracellular matrix interactions and paracrine stimuli [3,4,7-9].

Key to the study of GBM invasion is the availability of a reliable culture system, in order to preserve the tumorigenic potential of cells derived from patients, and of “in vivo” models suitable to address questions and test hypothesis concerning this process.

To these aims, we have successfully established long-term cell cultures from surgical tumor samples obtained from several GBM patients and demonstrated their ability to generate GBM xenografts by serial transplantation[10,11]. In particular, we observed that these cultures displayed two types of “in vivo” growth behavior in these transplants. The first one was mainly expansive while the second, causing the host’s white and gray matters substitution by tumor cells, was highly diffusive. The aim of the present study was to identify by microarray analysis if the two GBM culture types were characterized by differential gene expression. We actually identified a set of differentially expressed genes. Some of these were known to be involved directly or indirectly in promoting glioma invasion, which supported our results [12-14]. Other genes, however, were not previously described in association with glioma invasion. This study provides, therefore, a novel set of potential target genes for future research and development of treatment strategies intended to inhibit the invasion by GBM cells of healthy brain tissue.

## Methods

### Cell cultures

Long-term GBM cell cultures were obtained from surgical samples of tumors provided by the Neurosurgery Department of the San Martino Hospital in Genoa. An informed consent was obtained from all patients before surgery as required by the Ethic Board. Patients and tumors characteristics are provided in Table1.

**Table 1:** Patients, tumor characteristics and survival of mice inoculated with patient GBM cells

**Patient and GBM cells code**	**Gender**	**Age (years)**	**WHO**^**a**^**Glioma grade**	**Newly diagnosed/ recurrence**	**Tumor location**^**b**^	**Hemisphere**	**Lobe**	**Ventricular involvement**	**Meningeal infiltration**	**Survival (days) of mice bearing secondary tumors**
PT1	M	67	IV	new	cortical	right	temporal-occipital	No	No	55-65^c^
PT2	M	48	IV	new	cortico-undercortical	left	temporal-parietal-occipital	No	No	40-60^c^
PT3	M	53	IV	second recurrence	deep	left	parietal	Early subependymal involvement	Yes	80-90^c^
PT4	M	51	IV	new	undercortical	right	temporal-parietal	No	No	90-110^c^
PT5	F	70	IV	new	cortico-undercortical	right	frontal	No	No	90-110^c^
PT6	F	41	IV	new	cortico-undercortical	right	frontal-temporal	No	Yes	52-73

These cells generated serially transplantable tumors in vivo. Survival mice data represent range of days required to induce large brain tumors and the sacrifice of the animals after injection of 10^5^ GBM cells derived from explants of primary tumors (secondary tumors).

^a^World Health Organization (WHO) criteria for brain tumor classification.

^b^Determined by Nuclear Magnetic resonance.

^c^These data for the corresponding patient GBM long-term cell cultures were published by Griffero and collaborators [15].

Cells isolation was described in detail elsewhere [11,16]. Briefly, bioptic samples were plated in regular plastic dishes using proliferation medium. One to two weeks after plating, cellular aggregates, resembling neurospheres derived from normal neural precursors, were detectable in all glioblastoma cultures. Neurospheres-like aggregates were collected, dissociated to obtain a single cell suspension and seeded on Matrigel (BD Biosciences, San Jose, CA, USA) coated flasks. Under these conditions, cells grown as a monolayer gave rise to tumor when injected orthotopically in nude mice [11,16]. In addition, these long-term cultures of GBM tumorigenic cells maintained the ability to generate neurospheres-like aggregates repeatedly when transferred into flasks lacking Matrigel coating. In particular, the long-term cultures of GBM tumorigenic cells used in the present study did not show differences in growth pattern in vitro because they were all able to attach, spread and proliferate on Matrigel. Similarly, when seeded in culture flasks lacking the Matrigel coat, they were all able to generate neurosphere-like aggregates and proliferate with no major phenotype differences in neurosphere dimension and appearance (data not shown).

The proliferation medium was DMEM-F12/Neurobasal additioned with 1% v/v B27 supplement, (Gibco Ltd, Paisley, Scotland), 2 mM L-glutamine (Gibco Ltd), recombinant human fibroblast growth factor (FGF-2, 10 ng/ml Peprotech, London, UK), recombinant human epidermal growth factor (EGF, 20 ng/ml Peprotech). The medium was changed twice a week. Normal human astrocytes were purchased from ScienCell Research Laboratories (Carlsbad, CA) and cultured following the manufacturer’s instructions. Normalized expression levels for selected stem cell markers, nervous system markers, PDGF receptors and IDH genes in the tumorigenic long-term GBM cell cultures used in this study, are shown in Additional File 1: Table S1.

### Intracranial tumorigenicity assays

Human GBM cells in vivo tumorigenicity was tested by cell intracranial inoculation in 6 – 8-week-old NOD/SCID mice (Charles River Laboratories, Lecco, Italy).

NOD/SCID mice were housed in pathogen-free conditions, according to the National Regulation on Animal Research Resources. For intracranial inoculation, at least three mice for each GBM cell culture were used. Mice were anesthetized with i.m. ketamine and xylazine. Thereafter, the animal was positioned into a stereotactic frame (David Kopf Instruments, Tujunga, CA, USA) and a hole was made 2 mm lateral and 1 mm anterior from the bregma. Cells (10^5^) were injected using a Hamilton syringe (Sigma-Aldrich, Milan, Italy) at a depth of 3.5 mm in a vol of 2 μl. Mice were monitored for about 6 months for disease symptoms and were sacrificed by CO2 asphyxiation when they showed weight loss or any severe sign of disease. The brains of all sacrificed mice were collected and processed for histology. The survival in days of mice inoculated with the long-term GBM cultures used in this study is reported in Table1.

All experiments were performed in compliance with guidelines approved by the Ethical Committee for Animal Use in Cancer Research at the Istituto Nazionale per la Ricerca sul Cancro in Genoa, Italy. Under our experimental conditions, the minimum number of GBM cells required to give rise to tumor in mice was 10^4^.

### RNA extraction and quality control analysis

Total RNA was isolated from cultured GBM cells using miRNeasy® mini kit (Qiagen, Milan, Italy) with DNase treatment. RNA concentration and purity were determined by measuring absorbance at 260 and 280 nm; 2 μg total RNA was run on a 1% denaturing gel and 100 ng were loaded on the 2100 Bioanalyzer (Agilent, Palo Alto, CA) to verify RNA integrity.

### Amplification of RNA and array hybridization

According to the recommendations of the manufacturer, 100 ng of total RNA was used in the first-round synthesis of double-stranded cDNA. The RNA was reverse-transcribed using a Whole Transcript cDNA synthesis and amplification kit (Affymetrix UK Ltd., High Wycombe, UK). The resulting biotin-labeled cRNA was purified using an IVT clean-up kit (Affymetrix) and quantified using a UV spectrophotometer (A260/280; Beckman, Palo Alto, CA). An aliquot (15 μg) of cRNA was fragmented by heat and ion-mediated hydrolysis at 94°C for 35 minutes. The fragmented cRNA, run on the Bioanalyzer (Agilent Technologies, Santa Clara, CA) to verify the correct elettropherogram, was hybridized in a hybridization oven (16 hours, 45 °C) to a Human Gene 1.0 ST array (Affymetrix) representing whole-transcript coverage. Each one of the 28869 genes was represented on the array by approximately 26 probes spread across the full length of the gene, providing a more complete and more accurate picture of gene expression than the 3’ based expression array design. The washing and staining procedures of the arrays with phycoerythrin-conjugated streptavidin (Invitrogen, Monza, Italy) was completed in the Fluidics Station 450 (Affymetrix). The arrays were subsequently scanned using a confocal laser GeneChip Scanner 3000 7 G and the GeneChip Command Console (Affymetrix).

### GeneChip microarray analysis and data normalization

Affymetrix raw data files [cell intensity (CEL) files] were used as input files in expression console environment (Affymetrix). Briefly, CEL files were processed using the Robust Multi-Array Analysis (RMA) procedure [17], an algorithm that is publicly available at http://www.bioconductor.org. The RMA method was used to convert the intensities from the multiple probes of a probe set into a single expression value with greater precision and reduced background noise (relying on the perfect match probes only and thus ignoring the mismatch probes) and then to normalize by sketch quantile normalization. Quality assessments were also performed in the expression console environment. This procedure, based on various metrics, allowed us to identify a chip as an outlier (see for details Quality assessment of exon and gene arrays http://www.affymetrix.com/support/technical/whitepapers/exon_gene_arrays_qa_whitepaper.pdf). Significance Analysis of Microarrays (SAM), Principal Component Analysis (PCA) of variance and Hierarchical Clustering (HCL), after mean scaling and log2 transformation, were performed with the software tool from The Institute for Genomic Research (TIGR) MeV (multiple experimental viewer) (http://www.tigr.org/software/tm4/mev.html) [18].

Individual genes with different expression levels, among the two groups, were identified using SAM [19]. The false discovery rate expressed as q-value was used to evaluate statistical significance. For comparison purposes, an arbitrary filter was applied excluding all genes that did not exhibit a difference in expression of at least 2-fold. Genes differentially expressed were investigated using a two-class analysis.

We used PCA to reduce the complexity of high-dimensional data and to simplify the task of identifying patterns and sources of variability in these large data sets. The results from SAM were visualized using HCL [20]. All the microarray information has been submitted to the National Center for Biotechnology Information Gene Expression Omnibus web site (http://www.ncbi.nlm.nih.gov/geo/query/acc.cgi?token=zfutlaeiauqmmbo&acc=GSE16805).

### Pathways identification by Expression Analysis Systemic Explore (EASE)

Gene lists from Affymetrix results were examined using the EASE program, accessible via http://david.abcc.ncifcrf.gov/. EASE is a customized stand-alone software application with statistical functions for discovering biological themes within gene lists. This software assigns genes of interest into functional categories based on the Gene Ontology database (GO, http://www.geneontology.org/index.shtml) and uses the Fisher's exact test statistics to determine the probability of observing the number of genes within a list of interest versus the number of genes in each category on the array. A more detailed analysis of the genes' association with physiological pathways was performed using the Kyoto Encyclopedia of Genes and Genomes (KEGG, http://www.genome.jp/kegg/pathway.html). Each identified process was confirmed through PubMed/Medline (http://www.ncbi.nlm.nih.gov/sites/entrez?db=pubmed).

### RT-PCR analysis

Starting from about 1 μg of total RNA, cDNA was synthesized by using an Oligo(dT)20, random hexamers mix and a Superscript III first-strand synthesis system supermix for RT-PCR (Invitrogen). cDNAs, diluted 5–20 times, were then subjected to PCR analysis.

Relative quantification was performed by real-time quantitative RT-PCR (qPCR). Briefly, qPCR was performed and analyzed using the Mastercycler ep Realplex (Eppendorf AG, Hamburg, Germany). Primers were designed across a common exon–exon splice junction by using the tool available at https://www.roche-applied-science.com/sis/rtpcr/upl/index.jsp?id=UP030000 (Roche Applied Science, Monza, Italy)(see Additional File 2: Table S2). Reactions were carried out in triplicates and amplicons were measured by SYBR Green fluorescence (5 Prime, Hamburg, Germany) according to the manufacturer's recommendations. The dissociation curve analysis was used to define the specificity of the products by the presence of a single dissociation peak on the thermal melting curve.

The gene coding for the *peptidyl-prolyl cis-trans isomerase A* (*PPIA*) was used as the endogenous control for normalization because, in the microarray data, it showed in all conditions the steadiest expression in our experimental setting when compared with other housekeeping genes.

### Multiplex ligation-dependent probe amplification (MLPA) analysis

Genomic DNA was isolated from long-term GBM cell cultures and normal human astrocytes using QIAamp DNA microkit (Qiagen). The MLPA analysis (SALSA MLPA KITs P175-A1 Tumour-Gain and P294-A1 Tumour-loss, (MRC Holland, Amsterdam, the Netherlands) was performed using 100 ng of genomic DNA, diluted in 5 μl of TE buffer, following the manufacturer’s instructions. The resulting DNA fragments were identified and quantified by using capillary electrophoresis on an ABI XL3130 genetic analyzer (Applied Biosystems, Foster City, CA, USA) and the Genemapper program (version 4.0 - Applied Biosystems). Data were analyzed with the Coffalyser software (MRC-Holland). For each patient long-term culture of tumorigenic GBM cells, gains and losses were assigned by comparing the peaks between the patient and the reference samples (DNA from normal human astrocytes). A value =0 was considered as a biallelic loss, a value <0.7 corresponded to a DNA loss, a value between 1.3 and 2.0 corresponded to a DNA gain. The examined region was defined as amplified for values >2. All experiments were done in triplicate.

### Fluorescence in situ hybridization (FISH)

FISH was performed using a whole chromosome painting for chromosome 10 (WCP-10), obtained from Mariano Rocchi, Resources for Human Molecular Cytogenetics Project, by Telethon and the Italian Association for Cancer Research (AIRC). WCP-10 was amplified and labeled with Cy-dUTP (Amersham Pharmacia Biotech, Piscataway, NJ, USA) by degenerate oligonucleotide-primed polymerase chain reaction (DOP-PCR) [21]. Hybridization was performed as described [22], with minor modification. Briefly, slides carrying metaphase spreads were denatured in 70% (vol/vol) formamide/2x SSC, pH 7, at 70°C for 2 min], and dehydrated in a 4°C ethanol series. The hybridization mix consisted of 50% formamide, 2x SSC, 10% dextran sulfate, carrier DNA (sonicated salmon sperm DNA) at 500 μg/ml, human DNA C_0_t1 and Cy3-labeled WCP-10 at 2 μg/ml. This mixture was denaturated at 76°C for 6 min, incubated at 37°C for 30 min, applied to the slides under a glass coverslip, and sealed with rubber cement. After overnight incubation at 37^0^ C in Hybrite™ (Abbott Molecular/Vysis, Abbott Park, IL, USA), the slides were washed for 2 min at 72^0^ C in 0.4x SSC/0.3% NP40, pH 7 and for 1 min at R.T. in 2x SSC/0,1% NP40, pH7. Slides were counterstained with 4’,6- Diamidino-2-phenylindole, DAPI (Sigma-Aldrich) and mounted in Mowiol. Hybridization signals were evaluated using a digital image analysis system based on an epifluorescence (Provis AX70, Olympus, Milan, Italy), and images were acquired with a digital CCD camera C4742 Orca II (Hamamatsu, Japan) driven by Cytovision (Applied Imaging Corp., Santa Clara, CA, USA). The DAPI and Cy3 images were acquired with selective single-bandpass filters at 1000× optical magnifications.

### Immunofluorescence analysis

Antibodies for Sema5A (ab51957) were purchased from Abcam (Abcam, Cambridge, UK). The Nestin antibody (MAB353) was from Chemicon-Millipore (Millipore SPA, Milan, Italy). The BCAN antibody (HPA007865) was from Sigma-Aldrich. The Pmp2 antibody (12717-AP) was purchased from Protein Tech (Protein Tech Group, Inc., Chicago, IL, USA).

For xenograft tumor analysis, brains were cryopreserved and 10-μm cryostat (Leica Microsystems, Milan, Italy) sections were cut. Sections bearing tumors were identified by H&E. Cryosections containing tumors were permeabilized in PBS containing 0.2% Triton X-100 and blocked in 5% normal FCS-PBS. After incubation with primary antibodies (anti-Nestin), sections were stained with the appropriate secondary antibodies.

Cells plated on Matrigel-coated glass coverslips were fixed in 3% paraformaldehyde in PBS, pH 7.6 containing 2% sucrose for 5 min at room temperature. After PBS rinse, cells were permeabilized by a solution containing 20 mM Hepes pH 7.4, 300 mM sucrose, 50 mM NaCl, 3 mM MgCl_2_ and 0.5% Triton X-100 for 5 min on ice. Non-specific binding was prevented by incubation with pure goat serum for 30 min on ice. Slides were incubated with primary antibody in PBS supplemented with 10% goat serum or fetal bovine serum (antibody dilution buffer) for 2 hours on ice. After extensive PBS washes, slides were incubated for 30 min with the appropriate Alexa594 or Alexa488-conjugated secondary antibody in antibody dilution buffer. Nuclei were stained with Hoechst 33258 (Invitrogen).

Image acquisition were performed at 23°C with an Axiovert 200 M microscope equipped with the following filter sets: Zeiss 49, Zeiss 10 and Omega XF102-2 (Omega Optical, Brattleboro, VT, USA), which were used to detect Hoechst, Alexa 488 and Alexa 594 respectively.

### SDS PAGE and immunoblot analysis

Cell lysates were obtained as described previously [21]. SDS PAGE and immunoblotting were performed using precasted 4-12% polyacrylamide gels (Invitrogen) using manufacturer’s instructions. The blotting membranes were from Millipore (Millipore S.p.A., Vimodrone, Italy). The Pmp2 antibody (12717-AP) was purchased from Protein Tech (Protein Tech Group, Inc., Chicago, IL, USA). The antibodies for Bcan, Megf10, Pcdh10, Pcdh15 and Actin were all from Sigma-Aldrich (HPA007865, HPA026876, HPA011220, AV50153 and A 2066, respectively). The antibody for CD109 (Sc-271085) was purchased from Santa Cruz Biotechnology (Santa Cruz Biotechnology, Santa Cruz, CA, USA). The Gria2 antibody was acquired from Abcam ab40878.

## Results

### Human GBM cells in mouse orthotopic transplantation show two distinct patterns of tumor growth and tissue invasion

**Figure 1:**
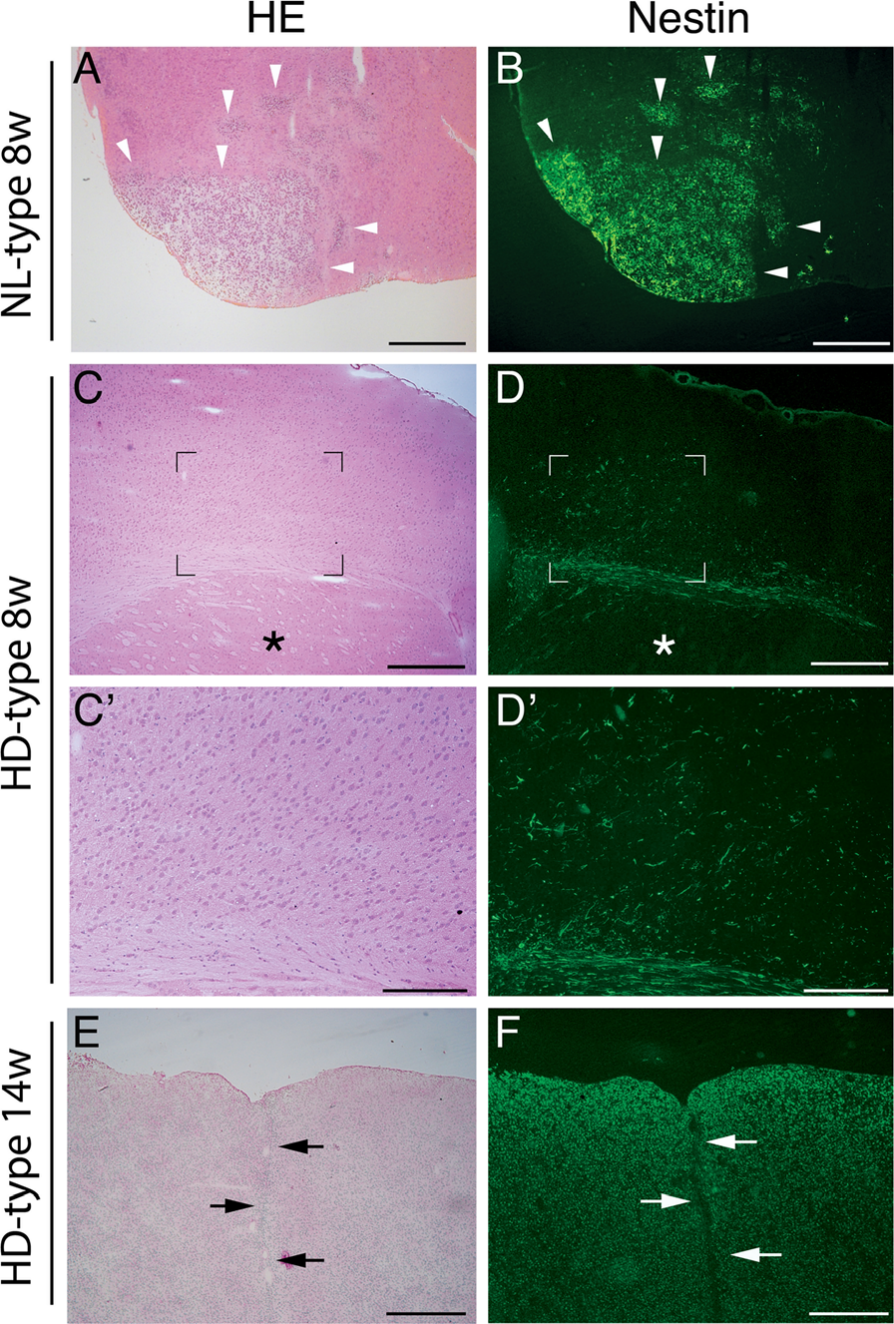
**Invasive behavior of human GBM cells in mouse orthotopic transplantation.** Hematoxylin and Eosin (HE) staining (**A**, **C**, **E**) and immunofluorescence analyses (**B**, **D**, **F**) of mouse brains injected with human cultured GBM cells. The immunofluorescence analyses were performed with an anti-human nestin antibody. Human nestin, yielding a green signal, identify GBM cells within the host tissue. Nodular-like growth pattern at 8 weeks post-injection, NL-type 8w (A, B; PT2). Highly diffuse and invasive growth pattern at 8 and 14 weeks post-injection, HD-type 8w (C, D; PT6) and HD-type 14w (E, F; PT6). The asterisks indicate the striatum in (C, D). Higher magnification images of the areas whose corners are indicated by four (L) in (C, D) are shown in (C’, D’). Arrowheads indicate tumor nodules in (A, B). Notice in (C, D) scattered human nestin-positive GBM cells and lack of nodules. Notice in (E, F) the complete substitution of the host’s tissue by the engrafted human nestin-positive GBM cells. Arrows in (E, F) indicate brain midline. Scale bar = 500 μm in (A-F). Scale Bar = 200 μm in (C’, D’).

In an effort to identify genes associated with the invasive properties of human GBM cells, we surveyed a collection of histologic samples of mouse brains obtained after orthotopic transplantation of GBM cell from long-term cultures, previously isolated from different patients (see Materials and Methods for details). This survey led us to identify two main patterns of behavior displayed by the tumors generated by these cultures in the host brain: nodular-like (NL-type), mostly expansive, mainly located in the subcortical regions of the injected hemisphere, and highly diffusive (HD-type) with a striking infiltrative phenotype. The HD-type GBM long-term cultures, in particular, used myelinated fibers to cross the midline and invade the hemisphere contralateral to the injection site and, with time, they finally substituted the host’s white and gray matter, including the cerebral cortex. For each transplanted brain, we stained contiguous sections with hematoxylin/eosin and an anti-human nestin antibody. This antibody allowed prompt identification of human GBM tumorigenic cells within mouse brains. Figure1 shows representative images of contiguous mouse brain sections hosting the two types of tumors generated by the GBM cultures. In the NL-type, the human GBM nestin-positive cells appeared to cluster together forming mainly nodules in sections, whereas in the HD-type, the human GBM nestin-positive cells scattered in the host brain mostly as single cells at both early and late stages of development (Figure1).

### Cultures of human tumorigenic GBM cells display unique chromosomal copy number aberrations unrelated to distinct in vivo invasion patterns

To investigate whether the two different in vivo invasion patterns of tumorigenic GBM cells used in the present study could be related to differences in gains or losses at genomic loci corresponding to known proto-oncogenes or tumor suppressor genes, we performed a Multiplex ligation-dependent probe amplification (MLPA) analysis. We investigated a total of 51 loci and found a number of alterations with respect to genomic DNA from primary cultures of human astrocytes, used a normal control reference. We observed that the cell cultures from each patient displayed a unique profile of losses and gains with the only exception for the CDKN2A biallelic loss, which was common to all six cultures (see Additional File 3: Table S3). However, we also observed that PT1, PT2 and PT3 (NL-type) cells shared a combined DNA gain at the EGFR locus and a DNA loss at the PTEN locus, which was not observed in the HD-type of GBM cells (see Additional File 3: Table S3). In fact, among HD-type of GBM cells we found either six copies of the EGFR locus or biallelic loss at the PTEN locus or no change at these two loci (PT4, PT6 and PT5, respectively, Additional File 3: Table S3). To investigate whether the two types of tumorigenic GBM cell cultures differed for chromosome 10 copy number, we performed a Fluorescence in situ hybridization (FISH) analysis using a whole chromosome 10-specific painting probe. The result of this analysis showed that chromosome 10 copy number cannot differentiate the two types of tumorigenic GBM cell cultures (Additional File 4: Table S4).

### Cultures of human tumorigenic GBM cells displaying distinct in vivo invasion patterns display a set of differentially expressed genes

We hypothesized that the different in vivo invasion pattern observed could be related to gene differentially expressed in the two groups of long-term tumorigenic GBM cultures. In order to identify these genes, we compared the gene expression patterns, obtained by microarray analysis performed on the Affymetrix platform. In particular, we used the TIGR MEV program and mRNAs extracted from GBM cultures previously isolated from patients 1–3 (PT1-3), belonging to the NL-type and from GBM cultures previously isolated from patients 4–6 (PT 4–6) belonging to the HD-type.

**Figure 2:**
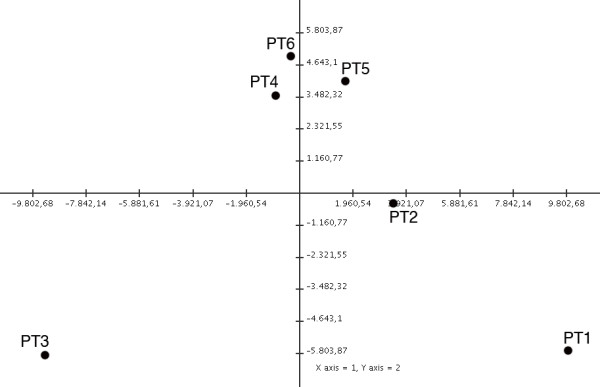
**Microarray analysis performed with TIGR MeV program: principal component analysis.** Microarray analysis of cultured human GBM cells generating tumor xenografts with nodular-like growth pattern, NL-type (PT1-3) or highly diffuse and invasive growth pattern, HD-type (PT4-6). Probe sets associated to dysregulation of gene expression levels among the six samples were identified using SAM (see Materials and Methods). Principal component analysis (PCA) is shown to provide the 2D projections onto the plane spanned by the two principal components for the data sets for each patient.

The principal component analysis (PCA), performed with the gene expression data, showed that samples belonging to the HD-type clustered together and could be promptly sorted from those belonging to the NL-type, possibly reflecting that HD-type samples were more homogeneous with respect to NL-types (Figure2). Each dot in the two-dimensional plot represented one sample. The distance between any pair of dots was related to the similarity between the two observations in high-dimensional space. Samples that were near each other in the plot were similar in a large number of variables, i.e., expression level of individual genes.

It should be noticed that the principal component analysis showed a scattering of the samples belonging to the NL-type (PT1-3). Based on gene expression, a molecular classification of glioblastoma into four different profiles called ProNeural, Neural, Classical and Mesenchymal was recently proposed [23]. For each of the 4 profiles, the corresponding centroid on the basis of the expression of 840 genes was identified. We extracted the expression profiles of these 840 genes from our dataset to classify our samples according to the correlation with each centroid. In this way we classified PT1, PT2 and PT3 as Neural, PT4 and PT6 as Mesenchymal (data not shown). Unfortunately, we were unable to similarly classify the profile of PT5. It is likely that PT5 was not similar to those previously published. Therefore, because PT1-3 were all Neural, their scattering in the principal component analysis appeared to result from the expression of genes different from those 840 used for the classification.

A complete list of 34 differentially expressed genes by a factor ≥ 2 in the comparison between the NL-type GBM cultures versus HD-type GBM cultures is provided (Table2). A short description of the known functions of these genes and their full names, as reported by http://www.genecards.org) [24], is shown in the Additional File 5: Table S5. 

**Table 2:** Probe sets ID, with relative mRNA Accession or Ensembl Transcript ID, gene symbols of genes regulated in the comparison between cultures of human GBM tumorigenic cells with the highly diffusive (HD-type) and the nodular-like (NL-type) invasive patterns, as determined by using SAM software with two-class unpaired analysis and the additional requirement of at least a 2-fold change in gene expression (Ratio)

	**Probe Set ID**	**mRNA Accession / ENSEMBL transcript ID**	**Gene Symbol**	**Ratio HD / NL expression**	q-value (%)^*^
1	8098021	NM_001083619	*GRIA2*	12.92	4.76
2	8144917	NM_000237	*LPL*	12.41	7.14
3	8151525	NM_002677	*PMP2*	10.90	4.76
4	7933672	NM_033056	*PCDH15*	10.68	4.17
5	7906205	NM_021948	*BCAN*	10.39	4.17
6	8168517	NM_005296	*LPAR4*	8.11	6.25
7	8097449	NM_032961	*PCDH10*	7.97	27.32
8	8107722	NM_032446	*MEGF10*	6.53	13.83
9	8135705	NM_012281	*KCND2*	6.36	22.98
10	8103736	NM_007281	*SCRG1*	6.14	0.00
11	8110932	NM_003966	*SEMA5A*	5.59	19.23
12	8065071	NM_198391	*FLRT3*	5.55	0.00
13	8146403	NM_018967	*SNTG1*	5.48	29.51
14	7954899	NM_001843	*CNTN1*	5.37	25.00
15	8055496	NM_018557	*LRP1B*	4.99	4.76
16	8095303	NM_015236	*LPHN3*	4.90	31.71
17	7947553	NM_020929	*LRRC4C*	4.83	5.56
18	8150978	NM_004056	*CA8*	4.60	0.00
19	8056457	NM_006920	*SCN1A*	4.51	3.70
20	8112881	ENST00000388321	*---*	4.48	29.51
21	8170307	NM_032539	*SLITRK2*	4.19	4.76
22	8162940	NM_005502	*ABCA1*	4.02	19.39
23	8082846	NM_004441	*EPHB1*	3.90	10.98
24	8037363	NM_145296	*CADM4*	3.88	25.17
25	7980580	NM_000153	*GALC*	3.57	25.00
26	7908812	NM_004767	*GPR37L1*	3.43	13.04
27	7970831	NM_007106	*UBL3*	3.22	10.98
28	8024712	NM_033064	*ATCAY*	2.98	4.17
29	8004081	NM_153018	*ZFP3*	2.96	4.76
30	8124280	NM_015864	*C6orf32*	0.36	3.57
31	7962579	BC095477	*AMIGO2*	0.29	3.45
32	8176375	NM_001008	*RPS4Y1*	0.21	20.80
33	8120719	NM_133493	*CD109*	0.18	20.72
34	8176719	NM_004681	*EIF1AY*	0.08	0.00

The false discovery rates are expressed as q-values^1^. Genes with GO term “cell adhesion”^#^ are shown in bold whereas genes with GO term “intrinsic to membrane”^§^ are shown with a gray background. A short description of the known functions of these genes and their full names as reported by http://www.genecards.org, are shown in the Additional File 5.

^*^The “q-value” is for each gene, the lowest False Discovery Rate at which that gene is called significant. It is like the well-known p-value, but adapted to multiple-testing situations.

^#^Gene Ontology cell adhesion definition: the attachment of a cell, either to another cell or to an underlying substrate such as the extracellular matrix, via cell adhesion molecules.

^§^Gene Ontology intrinsic to membrane definition: located in a membrane such that some covalently attached portion of the gene product, for example part of a peptide sequence or some other covalently attached group such as a GPI anchor, spans or is embedded in one or both leaflets of the membrane.

^*¶*^Likely overbalanced gene expression as chromosome Y-linked gene (see Table 1).

**Figure 3:**
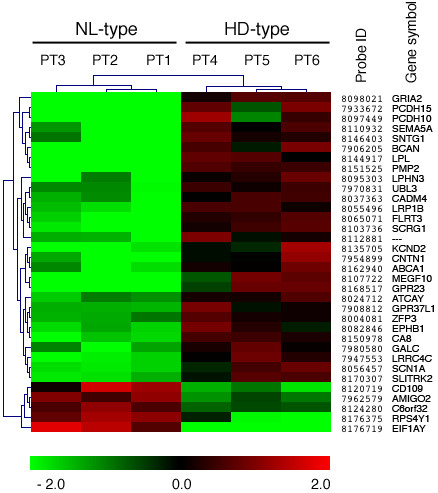
**Microarray analysis performed with the TIGR MeV program: hierarchical clustering.** Microarray analysis of cultured GBM cells and generating tumor xenografts with nodular-like growth pattern, NL-type (PT1-3) or highly diffuse and invasive growth pattern, HD-type (PT4-6). Heat map visualization obtained by hierarchical clustering (HCL). Probes corresponding to genes with similar regulation trend were placed close to each other as well as patient’s samples with overall similar gene expression pattern. The color-ratio bar at the bottom indicates intensity of gene up-regulation (red), down-regulation (green) and no change (black). Affymetrix Probes identification 7 digit numbers (Probe Id) along with Gene Symbols are shown on the right. Gene name symbols used are those approved by the Human Genome Organization Gene Nomenclature Committee (http://www.genenames.org/).

Differential expression of the genes included in Table2 was visualized by a heat map obtained by hierarchical clustering (HCL), which generates a tree (dendrogram) to group similar objects together (Figure3).

To gain a more mechanistic understanding of the processes associated to the HD phenotype, the EASE score [25] was used to identify Gene Ontology (GO) functional categories, which were significantly over-represented. After filtering the results, to avoid redundant and/or generic categories, two statistically significant GO term Biological Process and Cell Compartment were found associated with the HD-type of invasive phenotype: cell adhesion and intrinsic to membrane having a P-value of 1.1×10^-4^ and 1.9×10^-5^ respectively. In particular, 23 out of 34 genes were coding for intrinsic membrane protein and among these 23 genes, 9 were associated to cell adhesion processes (Table2).

### Validation at the protein level of the differential gene expression by NL-type and HD-type of human GBM tumorigenic cultures

**Figure 4:**
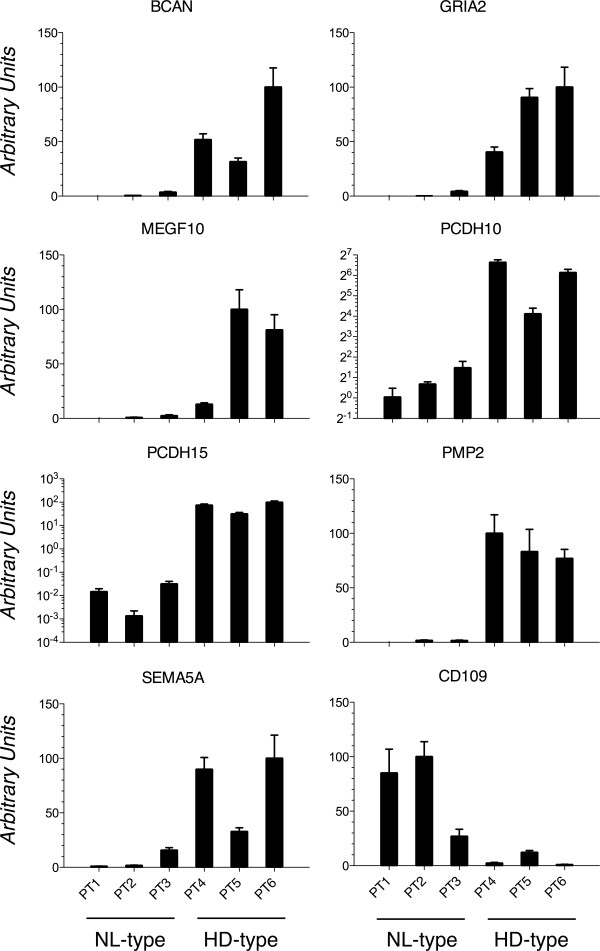
**Real-Time RT-PCR validation of microarray data.** Real-Time RT-PCR analysis performed on cultured GBM cells generating tumor xenografts with nodular-like growth pattern, NL-type (PT1-3) or highly diffuse and invasive growth pattern, HD-type (PT4-6) to validate the microarray data. This was accomplished on randomly selected genes from Table 2 and showed, in arbitrary units. Expression levels are relative to the expression of the housekeeping *peptidyl-prolyl cis-trans isomerase A *(*PPIA)* gene transcript. Standard deviations are indicated as vertical bars. Gene name symbols used are those approved by the Human Genome Organization Gene Nomenclature Committee (http://www.genenames.org/).

To validate gene expression changes, we performed Real time PCR analysis of arbitrarily selected genes (*BCAN, GRIA2, MEG10, PCDH10, PCDH15, PMP2, SEMA5A* and *CD109*) resulting as deregulated from the gene array experiments (Figure4). In particular, *BCAN, GRIA2, MEG10, PCDH10, PCDH15, PMP2 and SEMA5A* transcripts were found at least twice more abundant in the HD-type GBM cell cultures with respect to the NL-type whereas the *CD109* transcript was found down-regulated in HD-type with respect to NL-type GBM cell cultures as expected (Figure4).

**Figure 5:**
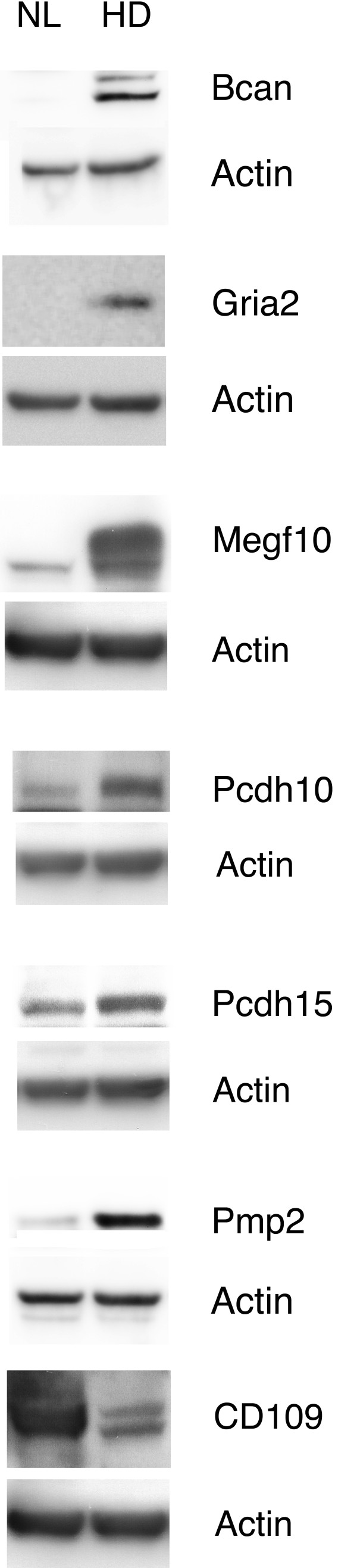
**Validation of gene expression regulation by Immunoblot analysis.** Western blot analyses were performed with lysates of cultured human GBM tumorigenic cells PT2 (belonging to the NL-type; NL) and PT6 (belonging to the HD-type; HD) challenged with Bcan, Gria2, Megf10, Pcdh10, Pcdh15, Pmp2 and CD109 antibodies. Each membrane was subjected to antibody stripping and rechallenged with an anti-actin antibody used as loading control.

**Figure 6:**
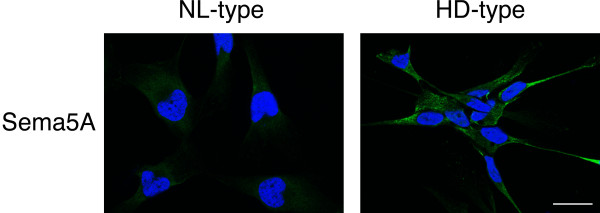
**Validation of gene expression regulation by Immunofluorescence analysis.** Indirect immunofluorescence analysis was performed on cultured human GBM tumorigenic cells PT2 (belonging to the NL-type) and PT6 (belonging to the HD-type), by using a specific antibody anti-Sema5A (green signal). Nuclei were stained by the Hoechst dye (blue signal). Merged imaged are shown. Scale bar = 20 μm,

In order to investigate whether the proteins encoded by the genes differentially expressed between HD- and NL-type GBM cells from the gene array experiments were actually expressed by these cells, we performed an immunoblot analysis focusing on those proteins encoded by the genes whose transcript levels in the two culture types were assayed by real time PCR analysis. We used commercially available antibodies for Bcan, Gria2, Megf10, Pcdh10, Pcdh15 and Pmp2 and CD109. We carried the immunoblot analysis on all the six GBM cell cultures used in the study revealing the expression of these proteins. Figure5 shows results obtained from a representative sample for each type of GBM cells, PT2 (NL-type) and PT6 (HD-type). Overall, we observed an agreement between the detected protein signal intensity and the level of transcript expression. Concerning the protein Sema5a, we performed an immunofluorescence analysis to evaluate the differential expression in the NL- and HD-type of GBM cells because the correspondent antibody was not suitable for immunoblot analysis. The immunofluorescenceanalysis result showed higher signal intensity for Sema5a in cells belonging to the HD-type with respect to cells belonging to the NL-type, which was similar to the signal obtained with a non-relevant control antibody (data not shown). Figure6 shows a representative experiment obtained with GBM cells from PT2 and PT6, NL- and HD-type, respectively, stained with the Sema5a antibody.

## Discussion

Targeting the GBM cell ability to invade the surrounding healthy CNS tissue is a goal to be obtained at the earliest stage of the disease in order to reduce the occurrence of relapses after surgery.

In order to contribute to the identification of novel target genes having a potential role in this process, we undertook a screening study by gene expression analysis.

We first observed that cells from our collection of GBM cultures, when transplanted in mice brains, were able to grow either as a nodule-like mass with a tendency to remain confined in the subcortical regions or to pervasively infiltrate as single cells the entire host’s CNS and eventually substitute the host’s tissue with the GBM tissue.

With this observation in mind, we hypothesized that specific genes able to drive or to inhibit the diffuse infiltrative behavior could be found up- or down-regulated respectively in GBM cultures displaying this in vivo phenotype.

Our gene expression analysis, by using principal component, first demonstrated that GBM cultures belonging to the diffuse infiltrating type clustered together separately from those belonging to the less infiltrating class. Secondly, cell adhesion appeared predominant among the functional processes associated to the 34 differentially expressed genes. Moreover, most genes belonging to this regulated set (23) appeared to encode for proteins intrinsic to the plasma membrane.

Real time PCR, immunofluorescence and immunoblot analyses, performed by using probes against eight arbitrarily selected gene products out of thirty-four, confirmed the differential regulation determined by microarray gene expression analysis.

Interestingly, among the genes that we found to be up-regulated in the HD-type, a few like *GRIA2*, *BCAN* and *LPAR4* were already shown to be directly or indirectly implicated with glioma cell invasion [12-14,26].

It is worth of note that because we did not observe a differential regulation of genes downstream of the EGFR, PI3K, P53 and AKT pathways, the combination of gains at the *EGFR* locus and losses at the *PTEN* locus, found in the NL-type and not present in the HD-type of cultures, was likely not responsible for the different in vivo invasion behavior. One of the possible explanations, for this lack of differential regulation of genes downstream of the mentioned pathways, is the finding of amplification at the *EGFR* locus (six copies) in PT4 and the biallelic loss at the *PTEN* locus in PT6 (both HD-type cells), which may result in stimulation of these pathways similar to the combination of monoallelic *EGFR* gain and *PTEN* loss.

Our data also showed that, although the long-term tumorigenic GBM cultures used in this study displayed loss of heterozygosis (LOH) at specific loci on chromosome 10q (i.e., *PTEN* and *RET*), they contained 2 copies (PT2, PT3, PT5, PT6) or 3 copies (PT1, PT4) of chromosome 10, independently from the in vivo invasion behavior. Therefore, monosomy for chromosome 10, which is frequent but not always observed in GBMs [27], could not be responsible for the observed different invasive property of these cells.

The difference in invasive behavior among HD-type and NL-typecould reflect in part the fact that two of the HD-type cultures were Mesenchymal (PT4 and PT6) whereas the NL-types were all Neural according to a GBM signature previously reported [23]. Interestingly, Tchoghandjian and collaborators, observed similar infiltrative and multifocal clusters in vivo invasion patterns displayed by Mesenchymal and Neural GBM-derived stem-like cells, respectively [28]. The result that *BCAN* was found expressed at the highest level in PT5 and PT6, which we classified as Mesenchymal, is in apparent disagreement with previous studies [23,29]. However, several reports showed that *BCAN* is associated to invasive glioma and promotes glioma invasion after proteolytic cleavage and fibronectin binding [12,14,30-34], which support our data. Moreover, the signature published by Verhaak and collaborators [23], which included *BCAN* and classified GBM cells, was based on the global expression of 840 genes. Therefore, in our opinion, some variation in the expression of a single gene may still occur without affecting GBM cell classification.

Previous studies have linked GBM cell lines in vitro and in vivo transplant growth patterns as well as gene signatures with cortical and deep tumor location in patient brains [28,29,35,36]. The analysis of the patient tumor characteristics, from which the long-term GBM cultures were established in our study, showed overall similarity in tumor location and lobe oforigin between the two invasive behavior types. A possible exception might be the frontal lobe involvement, which was present in two out of three patientsfrom which were derived the HD-type GBM cells and absent from those patients from which were established the NL-type of GBM cells. In our opinion, our series of cases is small to confirm or disprove a significant correlation between in vitro and in vivo growth patterns and invasive phenotype and tumor or patient characteristics, which was, however, not the aim of this study.

## Conclusions

In conclusion, we think that the present study has identified for the first time a set of genes that are likely to be implicated in the diffuse infiltration ability of GBM cells. In addition, most of the genes in this set encode for membrane proteins, which are, therefore, amenable to in vivo targeting approaches with conventional or recombinant antibodies.

## Competing interests

The authors declare that they have no competing interests.

## Authors’ contributions

MM participated in the design of the study, performed the real time RT-PCR, MLPA and immunoblot analyses, analyzed the data, performed the statistical analysis and wrote the manuscript. AD participated in the design of the study, isolated the GBM tumorigenic cells from bioptic materials, performed the in vivo study and analyzed the data. SC performed the immunofluorescence analysis. FR and VM performed the gene expression profile analysis. SV performed FISH analysis. SP contributed to analyze MLPA results. GZ and IM were responsible for patients’ management and provided clinical data. UP and WG analyzed the data. PC conceived the study, participated in the design of the study, analyzed the data wrote the manuscript. All authors read and approved the final manuscript.

## Pre-publication history

The pre-publication history for this paper can be accessed here:

http://www.biomedcentral.com/1471-2407/12/358/prepub

## Supplementary Material

Additional file 1 ** Table S1.** Normalized Expression levels for selected genes as determined by microarray analysis. Click here for file

Additional file 2 ** Table S2.** Sequences accession numbers and pri primers used for Real-Time PCR analysis. Click here for file

Additional file 3 ** Table S3.** Multiplex ligation-dependent probe amplification (MLPA) analysis. Click here for file

Additional file 4 ** Table S4.** FISH analysis of 50 metaphases for each cell line with WCP10 probe. Click here for file

Additional file 5 ** Table S5.** Gene symbol, full gene name and function of deregulated genes shown in Table 2. Click here for file
