# Supplementary material for: Identification of a novel set of genes reflecting different in vivo invasive patterns of human GBM cells
Source: BMC Cancer. 2012 Aug 17;12:358. doi: 10.1186/1471-2407-12-358 (PMC3502598; doi:10.1186/1471-2407-12-358)
Supplement: Additional file 1 — Table S1. Normalized Expression levels for selected genes as determined by microarray analysis. [file 1471-2407-12-358-S1.pdf]

Additional file 1. Normalized expression levels for selected genes as determined by microarray analysis

Stem cell markers

Probe Set ID	**PT1**	**PT2**	**PT3**	**PT4**	**PT5**	**PT6**	Gene Symbol	mRNA Accession
7970727	314.626635	298.269366	225.084515	272.437408	272.437408	272.437408	CDX2	NM_001265
7987365	104.918989	93.2385071	79.2542282	106.533991	100.733658	104.668535	NANOG	NM_024865
8124889	156.479299	147.00078	125.748293	147.00078	147.00078	147.00078	POU5F1	NM_002701
8084165	788.279803	1923.79586	2927.57416	3864.02845	2988.40507	3238.14079	SOX2	NM_003106
8015607	1312.98981	2095.32744	2639.94105	2991.72116	2026.70444	1971.78973	STAT3	NM_139276
8099476	40.3842824	147.727541	270.552545	51.9273969	57.2597315	363.494569	PROM1	NM_006017
7921088	490.320748	1543.79984	2099.65998	2127.12633	1589.77844	1738.34654	NES	NM_006617

Nervous System markers

Probe Set ID	**PT1**	**PT2**	**PT3**	**PT4**	**PT5**	**PT6**	Gene Symbol	mRNA Accession
7980580	114.736054	57.9670033	135.741495	324.544838	479.867069	295.958073	GALC*	NM_000153
8016128	276.637322	1101.84838	3275.5694	4939.87904	2121.48682	328.115969	GFAP	NM_002055
8047926	128.100562	1387.20953	1623.20556	3092.76178	3563.27966	3086.44421	MAP2	NM_002374
8072229	131.621011	115.282363	77.8014965	115.17829	115.282363	115.282363	NEFH	NM_021076
7998063	1147.54897	1105.3289	843.572936	1281.11121	1080.7971	1038.90642	TUBB3	NM_006086
7927482	204.115793	191.536429	134.546122	172.39164	175.210813	184.370241	CHAT	NM_020549
7945712	315.340574	258.091533	177.777262	225.01775	279.138633	253.748943	TH	NM_199292
8062539	215.946481	133.511296	134.245415	167.870044	155.361153	157.715976	SLC32A1	NM_080552
7938975	75.2493388	49.8919447	44.3598936	56.564461	57.543258	56.564461	SLC17A6	NM_020346
8038367	164.594464	140.662212	139.530142	140.662212	137.231037	140.305913	SLC17A7	NM_020309
8105220	158.04395	120.660863	98.9207999	120.660863	124.491463	115.453172	ISL1	NM_002202
8023889	149.859424	120.284172	85.7582761	105.517099	128.026531	109.623326	MBP	NM_001025101
8068231	158.807474	459.441383	605.612847	572.491713	530.344134	531.097526	OLIG2	NM_005806

PDGF receptor and IDH gene transcripts

Probe Set ID	**PT1**	**PT2**	**PT3**	**PT4**	**PT5**	**PT6**	Gene Symbol	mRNA Accession
8095080	4679.53674	558.105866	1739.94984	3898.78802	2349.81262	3655.2136	PDGFRA	NM_006206
8115099	226.347726	189.568047	258.072928	177.641765	240.329177	208.87022	PDGFRB	NM_002609
7985134	1489.70402	1113.51093	1195.7748	1412.9454	851.3243	780.04795	IDH3A	NM_005530
7991374	990.547261	1331.52149	1111.30569	1000.83193	1113.84286	1205.59531	IDH2	NM_002168
8058552	1838.95441	2815.56746	2803.90159	3831.57014	3516.19655	4648.17928	IDH1	NM_005896
8064522	1176.38127	1688.44562	1448.33538	1408.6134	1581.62296	1048.78991	IDH3B	NM_174856
8175844	879.742805	1218.34753	1264.89332	981.672506	892.637744	821.203519	IDH3G	NM_174869

*GALC is included in the 34 regulated genes (Table 2)
