# Supplementary material for: Identification of a novel set of genes reflecting different in vivo invasive patterns of human GBM cells
Source: BMC Cancer. 2012 Aug 17;12:358. doi: 10.1186/1471-2407-12-358 (PMC3502598; doi:10.1186/1471-2407-12-358)
Supplement: Additional file 2 — Table S2. Sequences accession numbers and pri primers used for Real-Time PCR analysis. [file 1471-2407-12-358-S2.pdf]

Additional File 2. Sequences accession numbers and primers used for Real-Time PCR analysis

Gene	Accession number	Forward (5'-3')	Reverse (5'-3')
BCAN	NM_021948	TTACGGAGACATGGATGGCTT	GGAGGGTCACCCAGGAACA
CADM4	NM_145296	GTCCAATAAGCACGGCCATG	GAACCGACGTCTGAGCCTCTA
EIF1AY	NM_004681	AGAGGTCTCACGAGGCTGTCA	CCCTGCGCCTGTTTTTACC
GRIA2	NM_001083619	CTGACACCCCACATCGACAA	AATAGCATAGACTCCTCTCGAAAACTG
PPIA	NM_021130	TTAGCCATGGTCAACCCCAC	GTCTTTGGGACCTTGTCTGCA
RPS4Y1	NM_001008	TGCTGTTCACCGCATCACA	CAGGTGAGGGATTCCCTTCA
SEMA5A	NM_003966	AGCCTCTGGCGTCCAAGTC	ACGCTACTGGATCTTGCCACA
