# Supplementary material for: Identification of a novel set of genes reflecting different in vivo invasive patterns of human GBM cells
Source: BMC Cancer. 2012 Aug 17;12:358. doi: 10.1186/1471-2407-12-358 (PMC3502598; doi:10.1186/1471-2407-12-358)
Supplement: Additional file 4 — Table S4. FISH analysis of 50 metaphases for each cell line with WCP10 probe. [file 1471-2407-12-358-S4.pdf]

Additional file 4. FISH analysis of 50 metaphases for each cell line with WCP10 probe.

**Chr.10 copies**	**1 (%)**	**2 (%)**	**3 (%)**	**4 (%)**
PT1	-	2 (4)	47 (94)	1 (2)
PT2 *	-	50 (100)	-	-
PT3	6 (12)	27 (54)	17 (34)	-
PT4	-	8 (16)	42 (84)	-
PT5	3 (6)	47 (94)	-	-
PT6	1 (2)	48 (96)	1 (2)	-

*In PT2 metaphases, besides the two chr 10 copies, some chr 10 material was present at the distal end of the p-arm of one (14% of the metaphases) or two (86% of the metaphases) derivative copies of an unknown chromosome.

For each cell line, a representative image is shown (FISH, left; DAPI-banding, right):

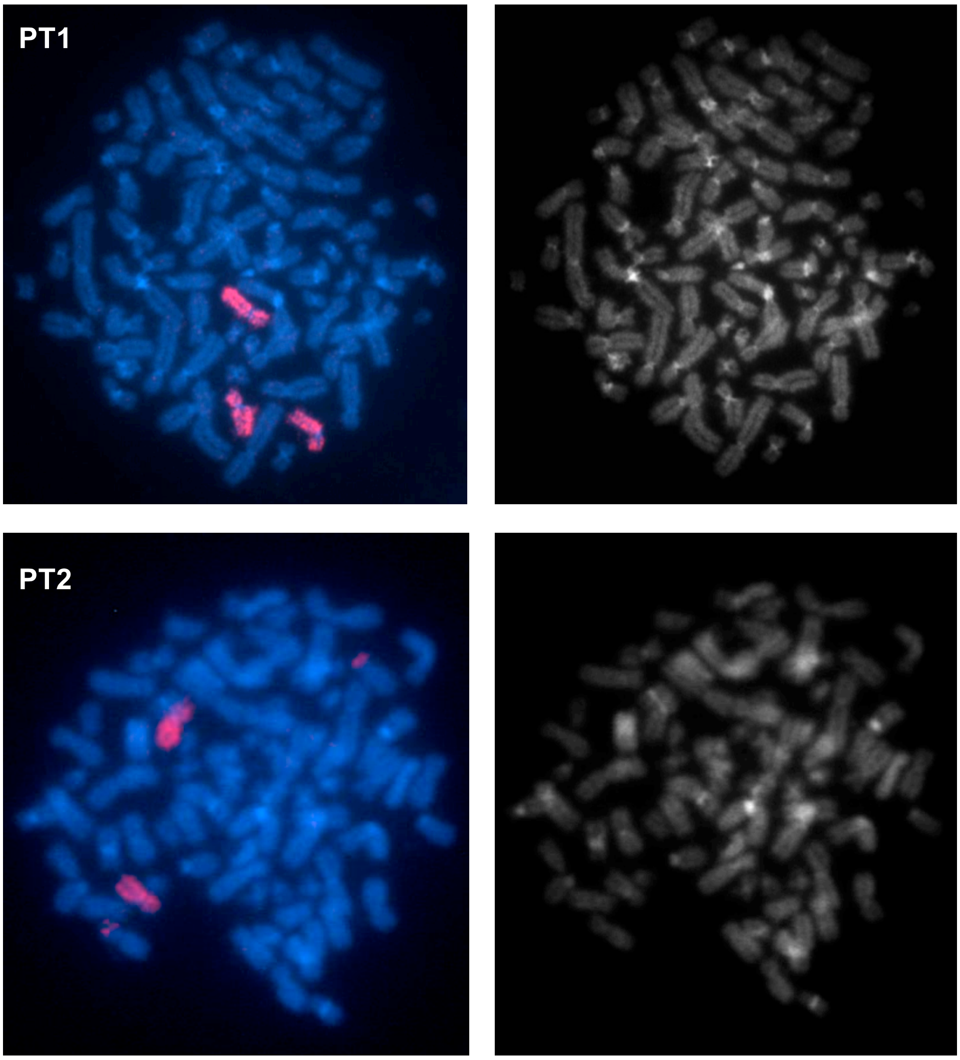

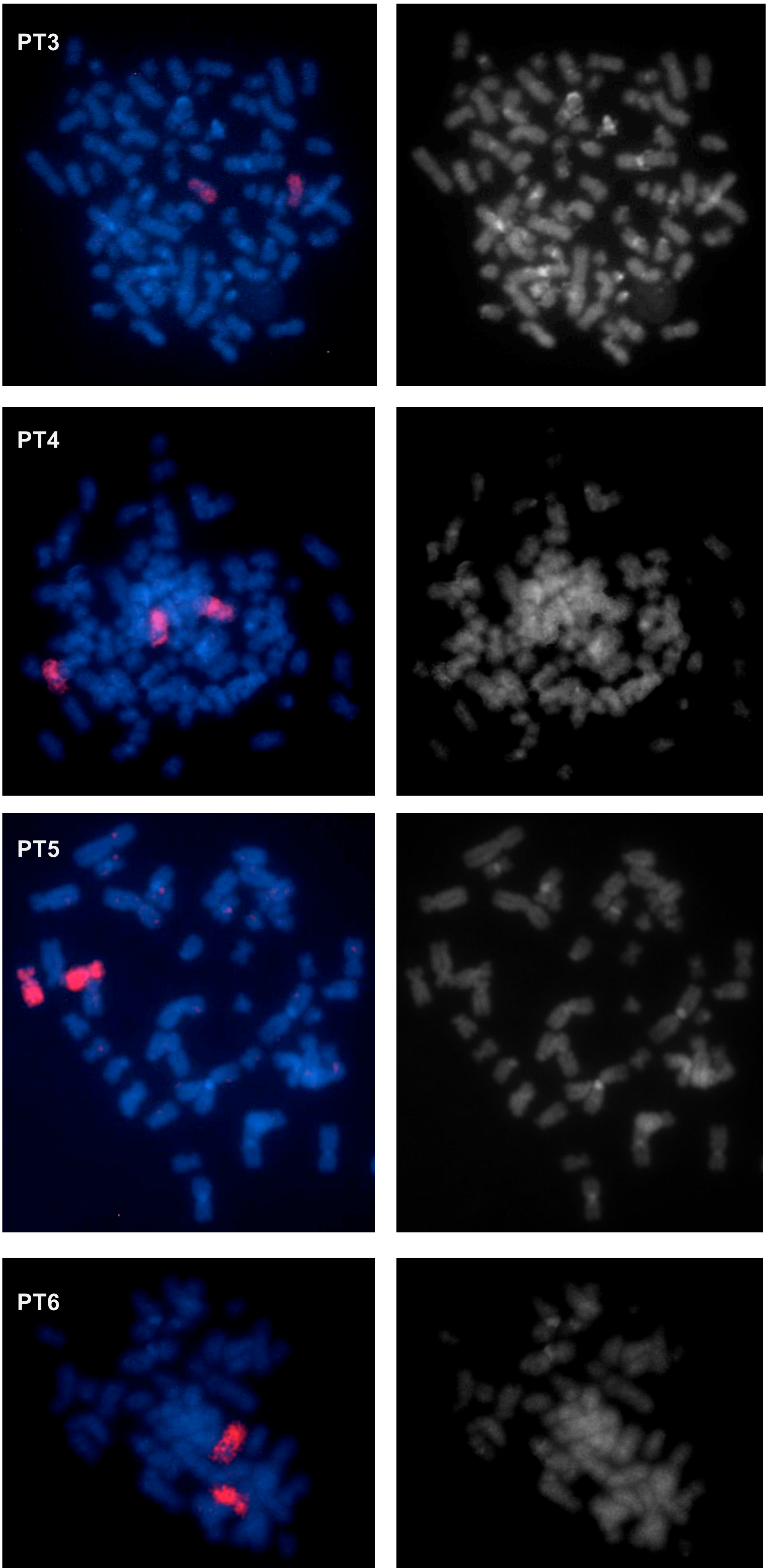
PT3
PT4
PT5
PT6
