# Supplementary material for: Identification of a novel set of genes reflecting different in vivo invasive patterns of human GBM cells
Source: BMC Cancer. 2012 Aug 17;12:358. doi: 10.1186/1471-2407-12-358 (PMC3502598; doi:10.1186/1471-2407-12-358)
Supplement: Additional file 3 — Table S3. Multiplex ligation-dependent probe amplification (MLPA) analysis. [file 1471-2407-12-358-S3.pdf]

Additional File 3. Multiplex ligation-dependent probe amplification (MLPA) analysis.

Gene	Band	PT1	PT2	PT3	PT4	PT5	PT6
DVL1 probe 12057-L04066	01p36.33	NC	NC	NC	NC	NC	NC
PRDM16 probe 04703-L04081	01p36.32	NC	NC	MAL	NC	NC	NC
CHD5 probe 09114-L09174	01p36.31	NC	NC	BAL	NC	NC	NC
CAMTA1 probe 04695-L13854	01p36.31	NC	NC	NC	NC	NC	NC
KIF1B probe 04681-L04462	01p36.22	NC	NC	NC	NC	NC	NC
MDM4 probe 03185-L03594	01q32.1	NC	NC	MAG	MAG	NC	MAG
MDM4 probe 03186-L02525	01q32.1	NC	NC	MAG	MAG	NC	MAG
MYCN probe 02572-L09025	02p24.3	NC	NC	NC	NC	NC	NC
MYCN probe 03327-L02466	02p24.3	NC	NC	NC	NC	NC	NC
ALK probe 08324-L08193	02p23.2	NC	NC	NC	NC	NC	NC
ALK probe 08323-L08192	02p23.2	NC	NC	NC	NC	NC	NC
VHL probe 01160-L00716	03p25.3	NC	NC	NC	NC	NC	NC
VHL probe 01161-L00717	03p25.3	NC	NC	NC	NC	NC	NC
FHIT probe 02290-L01781	03p14.2	NC	NC	NC	NC	NC	NC
FHIT probe 02293-L13849	03p14.2	NC	NC	NC	NC	NC	NC
PDGFRA probe 10247-L10719	04q12	NC	NC	NC	NC	NC	NC
PDGFRA probe 12762-L13878	04q12	NC	NC	NC	NC	NC	NC
KIT probe 12756-L14066	04q12	MAG	NC	NC	NC	NC	NC
KIT probe 12761-L13877	04q12	MAG	NC	NC	NC	NC	NC
KDR probe 12755-L14064	04q12	NC	NC	NC	NC	NC	NC
KDR probe 12758-L13874	04q12	NC	NC	NC	NC	NC	NC
DHFR probe S0428-L14095	05q14.1	NC	NC	NC	NC	NC	NC
DHFR probe 12753-L13869	05q14.1	NC	NC	NC	NC	NC	NC
APC probe 11990-L12817	05q22.2	NC	NC	NC	NC	NC	NC
APC probe 01550-L00993	05q22.2	NC	NC	NC	NC	NC	NC
EGFR probe 02063-L03283	07p11.2	MAG	MAG	MAG	AMPL	NC	NC
EGFR probe 06408-L05383	07p11.2	MAG	MAG	MAG	AMPL	NC	NC
MET probe 10314-L10828	07q31.2	MAG	MAG	NC	NC	MAG	NC
MET probe 10320-L10834	07q31.2	MAG	MAG	NC	NC	MAG	NC
SMO probe 12750-L13866	07q32.1	MAG	MAG	MAG	NC	MAG	NC
SMO probe 12757-L13873	07q32.1	MAG	MAG	MAG	NC	MAG	NC
BRAF probe 10507-L11060	07q34	MAG	MAG	NC	NC	MAG	NC
BRAF probe 04260-L14063	07q34	MAG	MAG	NC	NC	MAG	NC
FGFR1 probe 04184-L03583	08p11.23	NC	NC	NC	NC	NC	NC
FGFR1 probe 01046-L01837	08p11.23	NC	NC	NC	NC	NC	NC
MYC probe 00672-L00169	08q24.21	NC	AMPL	AMPL	NC	NC	NC
MYC probe 00580-L13856	08q24.21	NC	AMPL	AMPL	NC	NC	NC
CDKN2A probe 01528-L06031	09p21.3	BAL	BAL	BAL	BAL	BAL	BAL
CDKN2A probe 01524-L13846	09p21.3	BAL	BAL	BAL	BAL	BAL	BAL
CDKN2B probe 03814-L03851	09p21.3	BAL	BAL	BAL	BAL	BAL	BAL
PTCH1 probe 03709-L03163	09q22.32	NC	NC	NC	NC	MAG	NC
PTCH1 probe 09616-L13843	09q22.32	NC	NC	NC	NC	MAG	NC
ABL1 probe 12502-L13552	09q34.12	NC	NC	NC	NC	MAG	NC
ABL1 probe 12516-L13566	09q34.12	NC	NC	NC	NC	MAG	NC
TSC1 probe 04324-L03897	09q34.2	NC	NC	NC	MAG	MAG	NC
TSC1 probe 02351-L04302	09q34.2	NC	NC	NC	MAG	MAG	NC
TSC1 probe 04108-L13904	09q34.2	NC	NC	NC	MAG	MAG	NC
RET probe 05502-L04925	10q11.21	MAL	MAL	MAL	MAL	NC	NC
RET probe 05508-L13858	10q11.21	MAL	MAL	MAL	MAL	NC	NC
PTEN probe 06729-L06339	10q23.31	MAL	MAL	MAL	NC	NC	BAL
PTEN probe 03638-L02945	10q23.31	MAL	MAL	MAL	NC	NC	BAL
PTEN probe 03640-L02947	10q23.31	MAL	MAL	MAL	NC	NC	BAL
WT1 probe 02755-L13851	11p13	NC	NC	NC	NC	NC	NC
WT1 probe 02757-L14090	11p13	NC	NC	NC	NC	NC	NC
CCND1 probe 05401-L04807	11q13.2	NC	NC	NC	NC	NC	NC
CCND1 probe 05402-L04808	11q13.2	NC	NC	NC	NC	NC	NC
CCND1 probe 00583-L00148	11q13.2	NC	NC	NC	NC	NC	NC
CCND2 probe 03177-L02516	12p13.32	NC	NC	NC	MAL	NC	NC
CCND2 probe 00498-L00084	12p13.32	NC	NC	NC	MAL	NC	NC
CDK4 probe 03174-L12434	12q14.1	NC	NC	NC	NC	NC	NC
CDK4 probe 03173-L02512	12q14.1	NC	NC	NC	NC	NC	NC
MDM2 probe 07179-L06788	12q15	NC	AMPL	NC	NC	NC	NC
MDM2 probe 07180-L06789	12q15	NC	AMPL	NC	NC	NC	NC
BRCA2 probe 01599-L13847	13q13.1	MAL	NC	MAL	NC	NC	NC
BRCA2 probe 01603-L13850	13q13.1	MAL	NC	MAL	NC	NC	NC
RB1 probe 02583-L13845	13q14.2	MAL	NC	MAL	NC	NC	NC
RB1 probe 01798-L01361	13q14.2	MAL	NC	MAL	NC	NC	NC
MIRN15A probe 04019-L03416	13q14.3	MAL	NC	MAL	NC	NC	NC
DLEU1 probe 01062-L00639	13q14.3	MAL	NC	MAL	NC	NC	NC
TSC2 probe 01819-L13844	16p13.3	NC	NC	NC	NC	NC	NC
TSC2 probe 03168-L02571	16p13.3	NC	NC	NC	NC	NC	NC
TP53 probe 08785-L01159	17p13.1	MAL	NC	NC	NC	NC	NC
TP53 probe 02378-L13853	17p13.1	BAL	NC	NC	NC	NC	NC
TP53 probe 02262-L01496	17p13.1	NC	NC	NC	NC	NC	NC
AURKB probe 12749-L13865	17p13.1	MAL	NC	NC	NC	NC	MAG
AURKB probe 12759-L13875	17p13.1	MAL	NC	NC	NC	NC	MAG
NF1 probe 04074-L03710	17q11.2	NC	NC	NC	NC	NC	NC
NF1 probe 02519-L01950	17q11.2	NC	NC	NC	NC	NC	NC
ERBB2 probe 00675-L00572	17q12	NC	NC	NC	NC	NC	MAG
ERBB2 probe 00717-L00390	17q12	NC	NC	NC	NC	NC	MAG
TOP2A probe 01056-L14067	17q21.2	NC	NC	NC	NC	NC	MAG
TOP2A probe 01055-L00628	17q21.2	NC	NC	NC	NC	NC	MAG
BRCA1 probe 02821-L02250	17q21.31	NC	NC	NC	NC	NC	NC
BRCA1 probe 00830-L14092	17q21.31	NC	NC	NC	NC	NC	NC
BRCA1 probe 00827-L00342	17q21.31	NC	NC	NC	NC	NC	NC
SMAD4 probe 05143-L04533	18q21.2	NC	NC	NC	NC	MAL	NC
SMAD4 probe 07800-L07555	18q21.2	NC	NC	NC	NC	MAL	NC
STK11 probe 03124-L13848	19p13.3	NC	MAG	MAG	NC	MAG	MAG
STK11 probe 03126-L03339	19p13.3	NC	MAG	MAG	NC	MAG	MAG
FKBP8 probe 12754-L14091	19p13.1	NC	MAG	NC	NC	MAG	MAG
FKBP8 probe 12751-L13867	19p13.1	NC	MAG	NC	NC	MAG	MAG
AURKA probe S0429-L14094	20q13.31	NC	NC	NC	NC	MAG	NC
AURKA probe 10236-L14068	20q13.31	NC	NC	NC	NC	MAG	NC
SMARCB1 probe 08280-L08094	22q11.23	NC	NC	NC	NC	NC	NC
SMARCB1 probe 08295-L08109	22q11.23	NC	NC	NC	NC	NC	NC
FAM123B probe 12760-L13876	Xq11.1	NC	NC	NC	NC	NC	NC
FAM123B probe 12752-L13868	Xq11.1	NC	NC	NC	NC	NC	NC
AR probe 01690-L00423	Xq11.2	NC	NC	NC	NC	NC	NC
AR probe 12604-L14065	Xq11.2	NC	NC	NC	NC	NC	NC

BAL | MAL | NC | MAG | AMPL

The MLPA analysis was performed as outlined in the Materials and Methods section. For a prompt identification of the gene targeted by the oligonucleotide probes, an alternating yellow or white background was used for probe identification numbers showed in the Gene column. Similarly, in the cytogenetic band (Band) column, we used an alternating gray and white background to indicate probes targeting different chromosomal arms. For each patient long-term culture of tumorigenic GBM cells (PT1-PT6), gains and losses were assigned by comparing the peaks between the patient and the reference samples (DNA from normal human astrocytes). A value =0 was considered as a biallelic loss (purple, BAL), a value <0.7 as a (monoallelic) DNA loss (red, MAL), a value between 1.3 and 2.0 as a (monoallelic) DNA gain (green, MAG) and a value between 0.7 and 1.3 as unchanged with respect to the reference (NC, black). The examined region was considered as amplified for values >2 (light green, AMPL).
