# Supplementary material for: Identification of a novel set of genes reflecting different in vivo invasive patterns of human GBM cells
Source: BMC Cancer. 2012 Aug 17;12:358. doi: 10.1186/1471-2407-12-358 (PMC3502598; doi:10.1186/1471-2407-12-358)
Supplement: Additional file 5 — Table S5. Gene symbol, full gene name and function of deregulated genes shown in Table 2. [file 1471-2407-12-358-S5.pdf]

Additional File 5. Gene symbol, full gene name and function of deregulated genes shown in Table 2.

GeneSymbol	Description	mRNA Accession	Function (www.genecards.org)
ABCA1	ATP-binding cassette, sub-family A (ABC1), member 1	NM_005502	The membrane-associated protein encoded by this gene is a member of the superfamily of ATP-binding cassette (ABC) transporters. ABC proteins transport various molecules across extra- and intracellular membranes. ABC genes are divided into seven distinct subfamilies (ABC1, MDR/TAP, MRP, ALD, OABP, GCN20, White). This protein is a member of the ABC1 subfamily. Members of the ABC1 subfamily comprise the only major ABC subfamily found exclusively in multicellular eukaryotes. With cholesterol as its substrate, this protein functions as a cholesteral efflux pump in the cellular lipid removal pathway.
AMIGO2	Adhesion molecule with Ig-like domain 2	BC095477	Required for depolarization-dependent survival of cultured cerebellar granule neurons. May mediate homophilic as well as heterophilic cell-cell interaction with AMIGO1 or AMIGO3. May contribute to signal transduction through its intracellular domain. May be required for tumorigenesis of a subset of gastric adenocarcinomas
ATCAY	Ataxia, cerebellar, Cayman type	NM_033064	This gene encodes a neuron-restricted protein that contains a CRAL-TRIO motif common to proteins that bind small lipophilic molecules. Mutations in this gene are associated with cerebellar ataxia, Cayman type.
BCAN	Brevican	NM_021948	May play a role in the terminally differentiating and the adult nervous system during postnatal development. Could stabilize interactions between hyaluronan (HA) and brain proteoglycans
C6orf32 (FAM65B)	Chromosome 6 open reading frame 32	NM_015864	The protein encoded by this gene stimulates the formation of a non-mitotic multinucleate syncytium from proliferative cytotrophoblasts during trophoblast differentiation. Two alternatively spliced transcript variants have been found for this gene. Isoform 2 play a role in promoting myogenic cell differentiation, cytoskeletal rearrangement and filopodia formation.
CA8	Carbonic anhydrase VIII	NM_004056	he protein encoded by this gene was initially named CA-related protein because of sequence similarity to other known carbonic anhydrase genes. However, the gene product lacks carbonic anhydrase activity (i.e., the reversible hydration of carbon dioxide). The gene product continues to carry a carbonic anhydrase designation based on clear sequence identity to other members of the carbonic anhydrase gene family. The absence of CA8 gene transcription in the cerebellum of the lurcher mutant in mice with a neurologic defect suggests an important role for this acatalytic form.
CADM4	Cell adhesion molecule 4	NM_145296	Involved in the cell-cell adhesion. Has calcium- and magnesium-independent cell-cell adhesion activity. May have tumor-suppressor activity
CD109	CD109 molecule	NM_133493	This gene encodes a member of the alpha2-macroglobulin/complement superfamily. The encoded GPI-linked glycoprotein is found on the cell surface of platelets, activated T-cells, and endothelial cells. The protein binds to and negatively regulates signaling of transforming growth factor beta (TGF-beta).
CNTN1	Contactin 1	NM_001843	The protein encoded by this gene is a member of the immunoglobulin superfamily. It is a glycosylphosphatidylinositol (GPI)-anchored neuronal membrane protein that functions as a cell adhesion molecule. It may play a role in the formation of axon connections in the developing nervous system.
EIF1AY	Eukaryotic translation initiation factor 1A, Y-linked	NM_004681	This gene encodes a protein similar to eukaryotic translation initiation factor 1A (EIF1A). EIF1A is required for the binding of the 43S complex (a 40S subunit, eIF2/GTP/Met-tRNAi and eIF3) to the 5' end of capped RNA.
ENST00000388321	snoRNA_pseudogene	---	No protein product
EPHB1	EPH receptor B1	NM_004441	Receptor for members of the ephrin-B family. Binds to ephrin-B1, -B2 and -B3. Binding with the guidance cue ephrin-B2 at the optic chiasm midline redirect ventrotemporal (VT) retinal ganglion cells (RGCs) axons ipsilaterally. May be involved in cell-cell interactions in the nervous system
FLRT3	Fibronectin leucine rich transmembrane protein 3	NM_198391	This gene encodes a member of the fibronectin leucine rich transmembrane protein (FLRT) family. FLRTs may function in cell adhesion and/or receptor signalling. Their protein structures resemble small leucine-rich proteoglycans found in the extracellular matrix.
GALC	Galactosylceramidase	NM_000153	This gene encodes a lysosomal protein which hydrolyzes the galactose ester bonds of galactosylceramide, galactosylsphingosine, lactosylceramide, and monogalactosyldiglyceride. Enzyme with very low activity responsible for the lysosomal catabolism of galactosylceramide, a major lipid in myelin, kidney and epithelial cells of small intestine and colon.
LPAR4	Lysophosphatidic acid receptor 4	NM_005296	This gene encodes a member of the lysophosphatidic acid receptor family. It may also be related to the P2Y receptors, a family of receptors that bind purine and pyrimidine nucleotides and are coupled to G proteins. The encoded protein may play a role in monocytic differentiation.
GPR37L1	G protein-coupled receptor 37 like 1	NM_004767	Orphan receptor
GRIA2	Glutamate receptor, ionotropic, AMPA 2	NM_001083619	Ionotropic glutamate receptor. L-glutamate acts as an excitatory neurotransmitter at many synapses in the central nervous system. Binding of the excitatory neurotransmitter L-glutamate induces a conformation change, leading to the opening of the cation channel, and thereby converts the chemical signal to an electrical impulse. The receptor then desensitizes rapidly and enters a transient inactive state, characterized by the presence of bound agonist. In the presence of CACNG4 or CACNG7 or CACNG8, shows resensitization which is characterized by a delayed accumulation of current flux upon continued application of glutamate (By similarity)
KCND2	Potassium voltage-gated channel, Shal-related subfamily, member 2	NM_012281	Pore-forming (alpha) subunit of voltage-gated rapidly inactivating A-type potassium channels. May contribute to I(To) current in heart and I(Sa) current in neurons. Channel properties are modulated by interactions with other alpha subunits and with regulatory subunits.
LPHN3	Latrophilin 3	NM_015236	This gene encodes a member of the latrophilin subfamily of G-protein coupled receptors (GPCR). Latrophilins may function in both cell adhesion and signal transduction.
LPL	Lipoprotein lipase	NM_000237	Lipoprotein lipase (LPL), like LIPG, is a vascular lipase, however it is not synthesized in endothelial cells. It is anchored to the capillary endothelium by proteoglycans and catalyzes the hydrolysis of triglycerides to release free fatty acids into the circulation. LPL therefore initiates the processing of triglyceride-rich lipoproteins such as chylomicrons and VLDL.
LRP1B	Low density lipoprotein receptor-related protein 1B	NM_018557	LRP1B belongs to the low density lipoprotein (LDL) receptor gene family. These receptors play a wide variety of roles in normal cell function and development due to their interactions with multiple ligands.
LRRC4C	Leucine rich repeat containing 4C	NM_020929	NGL1 is a specific binding partner for netrin G1, which is a member of the netrin family of axon guidance molecules.
MEGF10	Multiple EGF-like-domains 10	NM_032446	Involved in phagocytosis by macrophages of apoptotic cells. Cooperates with ABCA1 during engulfment. Destabilizes the oligomeric assemblies of the ABCA1 transporter. Plays a role in cell adhesion. Inhibits cell motility and cell proliferation in vitro. Promotes formation of large intracellular vacuoles.
PCDH10	Protocadherin 10	NM_032961	This gene belongs to the protocadherin gene family, a subfamily of the cadherin superfamily. The mRNA encodes a cadherin-related neuronal receptor thought to play a role in the establishment and function of specific cell-cell connections in the brain.
PCDH15	Protocadherin-related 15	NM_033056	This gene is a member of the cadherin superfamily. Family members encode integral membrane proteins that mediate calcium-dependent cell-cell adhesion. It plays an essential role in maintenance of normal retinal and cochlear function.
PMP2	Peripheral myelin protein 2	NM_002677	May play a role in lipid transport protein in Schwann cells. May bind cholesterol.
RPS4Y1	Ribosomal protein S4, Y-linked 1	NM_001008	Ribosomal protein S4 is the only ribosomal protein known to be encoded by more than one gene, namely this gene and ribosomal protein S4, X-linked (RPS4X). The 2 isoforms encoded by these genes are not identical, but are functionally equivalent.
SCN1A	Sodium channel, voltage-gated, type I, alpha subunit	NM_006920	Sodium channel, voltage-gated, type I, alpha subunit
SCRG1	Stimulator of chondrogenesis 1	NM_007281	Scrapie-responsive gene 1 is associated with neurodegenerative changes observed in transmissible spongiform encephalopathies. It may play a role in host response to prion-associated infections.
SEMA5A	Sema domain, seven thrombospondin repeats (type 1 and type 1-like), transmembrane domain (TM) and short cytoplasmic domain, (semaphorin) 5A	NM_003966	This gene belongs to the semaphorin gene family that encodes membrane proteins containing a semaphorin domain and several thrombospondin type-1 repeats. Members of this family are involved in axonal guidance during neural development.
SLITRK2	SLIT and NTRK-like family, member 2	NM_032539	This gene encodes an integral membrane protein that contains two N-terminal leucine-rich repeats domains and contains C-terminal regions similar to neurotrophin receptors. The encoded protein may play a role in modulating neurite activity.
SNTG1	Syntrophin, gamma 1	NM_018967	Adapter protein that binds to and probably organizes the subcellular localization of a variety of proteins. May link various receptors to the actin cytoskeleton and the dystrophin glycoprotein complex (By similarity). May participate in regulating the subcellular location of diacylglycerol kinase-zeta to ensure that diacylglycerol is rapidly inactivated following receptor activation.
UBL3	Ubiquitin-like 3	NM_007106	**No information is provided.**
ZFP3	Zinc finger protein 3 homolog (mouse)	NM_153018	May be involved in transcriptional regulation.
